# Data-Driven Learning of Optimal Position-Dependent Exact-Exchange Energy Density Mixing for Improved Density Functionals

**DOI:** 10.1021/acs.jpca.5c06845

**Published:** 2025-12-31

**Authors:** Martin Kaupp, Nóra Kovács, Artur Wodyński

**Affiliations:** Institut für Chemie, Theoretische Chemie/Quantenchemie, 26524 Technische Universität Berlin, Sekr. C7, Straße des 17. Juni 135, D-10623 Berlin, Germany

## Abstract

A transparent, data-driven route to improve approximate density functionals by learning only the position dependence of an exact-exchange admixture in local hybrid functionals is discussed. Motivated by the scarcity of exact constraints for valence regions and by gauge ambiguity issues of exchange-energy densities, we replace hand-crafted inhomogeneity measures by neural-network local mixing functions (n-LMFs) evaluated on rung-3 or rung-4 descriptors while keeping the overall structure of the functional transparent and explainable. This limited use of machine learning (ML) has already provided a number of practical outcomes. The LH24n-B95 and LH24n functionals achieve broad main-group accuracy and, strikingly, suppress gauge artifacts without use of so-called calibration functions. The reasons for the latter observation can be visualized and analyzed directly in real space. Extending the idea to rung-5 functionals leads to the first local double hybrids in which a position-dependent exact-exchange admixture is paired with an SCS-PT2 correlation, delivering consistent gains over constant-mixing analogues. To escape the zero-sum game between delocalization errors and static-correlation errors, an n-LMF has subsequently been trained in the presence of an explicit strong-correlation factor. The resulting LH25nP functional combines the so far best rung-4 performance for main-group energetics with improved spin-restricted bond-dissociation curves, fractional-spin behavior, and reduction of spin-contamination artifacts, while remaining numerically robust. The limited ML approach preserves explainability and facilitates the transfer of insights back to rational designs.

## Graphical abstract

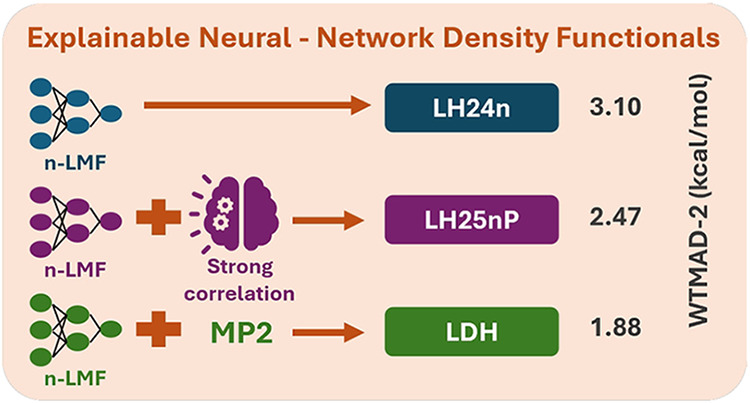

## 1 Introduction

Kohn–Sham density functional theory (KS-DFT) is currently the workhorse in electronic structure theory, from quantum chemistry to solid-state physics, as the scaling of its computational effort with system size is more favorable than that of accurate many-body methods.^1–6^ The success of KS-DFT is closely coupled to the quality of the approximate exchange-correlation (XC) functional employed in a given computation. The main challenges in improving the accuracy of density functional approximations (DFAs) are (a) reducing self-interaction errors (often termed delocalization errors),^7–10^ (b) reducing static-correlation errors,^11,12^ and (c) simulating dispersion interactions. With a number of approaches available to deal quite accurately with the latter aspect, at least for nonmetallic systems,^13–15^ the trade-off between the first two aspects has become the central focus in DFA development.^6,16–19^ That is, in conventional global or range-separated hybrids with a single, system-independent fraction of exact (“Hartree–Fock-like”) exchange (EXX), increasing the EXX admixture to reduce delocalization/self-interaction errors (e.g., in barrier heights, charge-transfer excitations, or band gaps) simultaneously diminishes the semilocal exchange contribution that partly mimics left–right correlation, thereby worsening static-correlation errors in stretched bonds and diradicals. This pervasive empirical trade-off between delocalization and static-correlation errors has often been described as a “zero-sum game” ^16,17^ in DFA design, and local-hybrid approaches explicitly aim at escaping this zero-sum behavior by using the exact-exchange energy density as a local mixing indicator.^6^

Among the approaches toward escaping this zero-sum game, we have recently emphasized DFAs based on the EXX energy density in coordinate space.^6^ Coordinate-space approaches for strong-correlation contributions have been pioneered in Becke’s B13 model,^20^ adapted also in the KP16/B13 scheme of Kong and Proynov.^21^ We have shown that these ideas can be transferred successfully to more routinely usable, widely applicable DFAs within the framework of local hybrid (LHs) functionals.^6,22–24^ Going beyond the widely used standard “global hybrids” (GHs) with constant EXX admixture,^25,26^ or so-called range-separated hybrids (RSHs) that vary EXX admixture along the interelectronic distance coordinate,^27,28^ LHs use a real-space position-dependent EXX admixture governed by a local mixing function (LMF).^29^ We have shown that strong-correlation factors inspired by the B13 and KP16/B13 ideas help LHs significantly improve the spin-restricted dissociation of covalent bonds.^6,22–24^ These “scLHs” also improve on other aspects related to static correlation, e.g., they reduce unphysical spin-symmetry breaking in open-shell transition-metal complexes^30^ or improve magnetic properties of systems with appreciable static correlation.^31^ Our currently most universal and accurate functionals combine the LH and RSH ideas in the form of range-separated local hybrid functionals (RSLHs).^32^ When using full long-range EXX admixture, RSLHs can provide the correct asymptotic XC potential and provide particularly accurate quasi-particle energies and/or performance for charge-transfer excitations in time-dependent DFT (TDDFT) calculations.^33,34^ When including correction terms for strong correlation as well as for delocalization errors in “abnormal open-shell regions” (inspired by the PSTS functional^35^) such scRSLHs,^18^ like the most recent ωLH25tdE,^19^ provide the currently most substantial escape from the zero-sum game for practically applicable functionals. We note in passing that on the more flexible but computationally more demanding rung-5 of the functional ladder, developments toward escaping the zero-sum game are also promising and actively pursued.^36–39^

The scLHs and scRSLHs described above were based on LMFs constructed by using heuristic inhomogeneity functions. Often the starting point has been a so-called “t-LMF”, i.e., a scaled ratio of von-Weizsäcker and Kohn–Sham kinetic-energy densities.^29,40,41^ Then, strong-correlation factors further modify the t-LMFs in spatial regions, where our detection functions for static correlation become significant. Such LHs and RSLHs, as well as their scLH and scRSLH extensions, have been very successful. However, t-LMFs have their shortcomings, both regarding exact constraints and their tendency^42^ to exacerbate the so-called gauge problem that arises due to the ambiguity of exchange-energy densities (see, e.g., ref 29 and references therein). In fact, only a few exact constraints on LMFs are known, and we had previously considered both these constraints and some “desirable shape features” in our constructions.^29^ What is known is the exact behavior in one-orbital regions, as well as the coordinate scaling condition in the high-density limit, but all of these constraints pertain to either regions near heavy nuclei or to the asymptotic regions far from the nuclei. No constraints are known regarding the optimum behavior of LMFs, i.e., of the position dependence of the EXX admixture of LHs or RSLHs, in the valence and bonding regions of molecules or solids. We note in passing that this is consistent with the general observation that insufficient physical constraints are available to define functionals on rung-4 of the usual ladder hierarchy.^43^

Unless more exact physical constraints on the valence-space behavior become available, this points to the need for a data-driven approach. Machine-learning approaches to the development of DFAs have exploded during the past decade, and the literature has already become too extensive to be discussed here in detail.^44–56^ An important development pertinent to this perspective is Google Deep Mind’s DM21 functional,^46^ which is a deep-neural network (NN) functional trained, among other things, on specific data sets incorporating fractional-charge and fractional-spin behavior to reduce delocalization and static-correlation errors, respectively. In view of the input features to the deep-neural network, DM21 can also be considered an scRSLH, but the completely black-box nature of the functional does not allow for further insights. DM21 performs well and escapes the zero-sum game to a significant extent, but so far, it does not seem to be a production-level functional. Its implementation does not appear very efficient, and it has been criticized, e.g., regarding SCF convergence for transition-metal systems.^57^ We also note the Skala functional reported recently by Microsoft Research,^56^ which is a deep-neural network functional that is more computationally efficient than DM21 due to its lower-rung input features, but which does not cover strong correlations. Skala is also essentially a black box. A different application of machine learning (ML) to train EXX admixture in hybrid functionals should also be mentioned, i.e., to learn a system-dependent constant admixture in GHs^58^ or a system-dependent range separation parameter in “tuned” RSHs.^59–61^ The disadvantage of the system-dependent parametrization of such approaches lies in having a potentially different functional for each molecule. This causes an ambiguity for, e.g., reactions between components having different optimal parameters, which is something we can avoid with LHs or RSLHs.

This feature article deals with our own recent efforts to utilize machine learning in a more limited but also much more transparent way in the context of training the LMF of LHs. This has already led to the first LHs with a neural-network LMF (n-LMF) without strong-correlation factors (LH24n-B95 and LH24n),^62^ to the first (range-separated) local double hybrids (LDHs),^63^ and most recently to the first scLH with an n-LMF and strong-correlation factors, LH25nP.^64^ Here we report on the insights and numerical findings obtained during these endeavors and provide an outlook on possible future directions for DFA development.

## 2 Brief Theoretical Background of LHs and of Coordinate-Space Models of Strong Correlation

The background of LHs has been reviewed in 2019,^29^ before sc-factors had been introduced. We also refer the reader to a relatively recent account article on scLHs and scRSLHs.^6^ Here, we only delineate some essential aspects that help us appreciate the subsequent development of machine-learned LMFs. The best way to understand the mechanism by which an LH can simulate static correlation is to write it as

1 $$E_{\mathrm{XC}}^{\mathrm{LH}}=E_{X}^{\mathrm{ex}}+\int \left(1-g\left(r\right)\right)\left(e_{X}^{\mathrm{sl}}\left(r\right)-e_{X}^{\mathrm{ex}}\left(r\right)+G\left(r\right)\right)dr+E^{\mathrm{DC}}$$

Here, we add to full EXX (first term) the important middle term that essentially is supposed to mimic nondynamical correlation (NDC) and the last term largely responsible for dynamic correlation (DC). We see that the integrand of the middle term contains the complement, i.e., 1 – *g*(**r**), of the LMF *g*(**r**). This complement multiplies the difference between semilocal and exact exchange-energy densities. Those energy densities are only defined up to a “calibration function” (CF) *G*(**r**) that integrates to zero over all of space. Specifically designed CFs^65,66^ had to be added to previous t-LMF-based LHs and RSLHs to mitigate unphysical NDC contributions arising from the gauge ambiguity. The latter manifested in particular in too repulsive potentials in the context of noncovalent interactions (NCIs), and this has been used to parametrize CFs, e.g., in the LH20t^67^ or ωLH22t^33^ functionals, to mitigate the gauge problem. We will come back to this point later.

The structure of Becke’s B05 coordinate-space model of NDC^68^ is closely analogous to eq 1 and its three contributions. In contrast to an LH, the middle term of B05 is, however, based on the addition of further EXX contributions to deepen the exchange-correlation hole in regions where comparisons between hole normalizations of the EXX and a model hole (“reverse Becke–Roussel machinery,”^68^ revBR) indicate the necessity. However, Becke noted that this middle term is not able to recover the strong correlations found when stretching chemical bonds appreciably or to reduce fractional-spin errors.^20^ He argued that the B05 middle term covers only the potential energy of static correlation and misses the kinetic-energy correlation contribution. To recover the latter from interpolation along a local version of the adiabatic connection, he constructed the B13_str_ term and added it to the functional form.^20^

Our transfer of such ideas to the LH framework started from the KP16/B13 model of Kong and Proynov,^21^ who multiplied the integrand of the middle term of a B13-type expression by a strong-correlation factor *q*_AC_(**r**). Equation 2 shows the form of an scLH obtained along these lines. *q*_AC_(**r**) is 0.5 in the absence of strong correlations but might approach 1.0 locally in strong-correlation situations. In the KP16/B13 model and in our initial scLH constructions, *q*_AC_(**r**) was based on the same revBR machinery based on hole normalizations. In our more recent scLH models,^18,24,64^ we used simple ratios $z\left(r\right)=\frac{e_{X}^{\mathrm{sl}}}{e_{X}^{\mathrm{ex}}}$ between semilocal and exact exchange-energy densities as real-space detection functions for static correlations. Notably, *q*_AC_(**r**) becomes part of the final, modified LMF. One can see from eq 2 that by locally diminishing EXX admixture in the overall LMF *g*(**r**), *q*_AC_(**r**) actually enhances the simulation of static correlation. Indeed, Figure 1 shows that upon spin-restricted stretching of a covalent bond, the final sc-corrected LMF may become locally negative during bond dissociation. This is part of the mechanism by which *q*_AC_(**r**) helps to improve the description of bond dissociation (Figure 1). In the conventional case 0 ≤ *g*(**r**) ≤ 1, *g*(**r**) = 1 corresponds locally to pure exact exchange, whereas *g*(**r**) = 0 corresponds to pure semilocal exchange. Negative values of the final LMF in an scLH during bond dissociation enhance the semilocal contributions and diminish the EXX contributions further. In close analogy to the philosophy of the B13 and KP16/B13 coordinate-space models of strong correlations,^20,21^ this simulates additional kinetic-energy contributions to static correlation obtained from a local adiabatic connection.^22,23^ We note that in eq 2, *q*_AC_(**r**) also adiabatically connects the DC contribution. This has been found empirically to give superior results in some constructions. We will come back to this further below.

2 $$E_{\mathrm{XC}}^{\mathrm{scLH}}=E_{X}^{\mathrm{ex}}+\int 2q_{\mathrm{AC}}\left(z\left(r\right)\right)\{\left(1-g\left(r\right)\right)\left[e_{X}^{\mathrm{sl}}\left(r\right)-e_{X}^{\mathrm{ex}}\left(r\right)+G\left(r\right)\right]+e_{C}^{\mathrm{DC}}\left(r\right)\}dr$$

**Figure 1:**
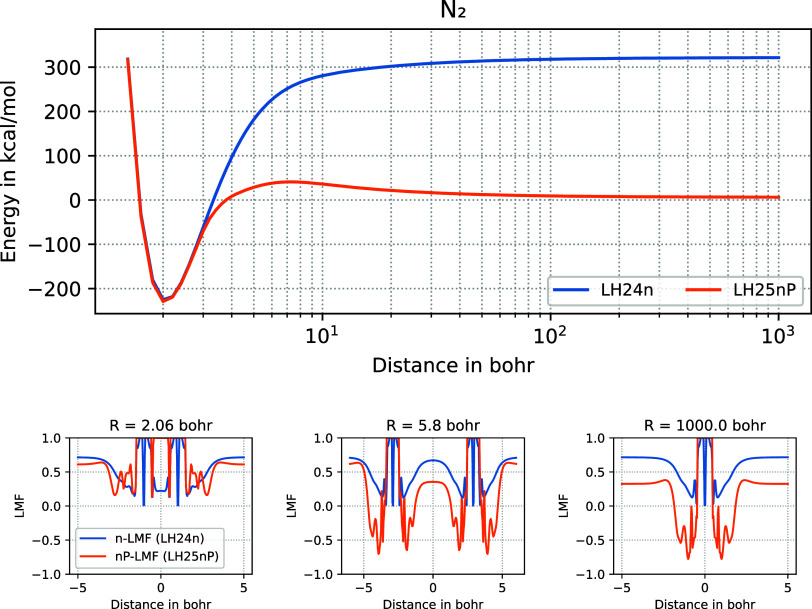
Spin-restricted dissociation curves of N_2_ obtained with LH25nP and LH24n for comparison (top) and the corresponding LMFs at different internuclear distances (lower panel). At 1000 b, only one of the two nitrogen atoms is inside the LMF-plot. See text for a discussion on how reduced or even negative EXX admixture in the LMF upon bond stretching is linked to the recovery of strong correlations and the improvement of the dissociation asymptote.

A transfer of such strong-correlation terms to an RSLH framework is more involved as we also have to touch the range separation aspect. Notably, in an RSLH with full long-range EXX admixture, the integrand of the NDC middle term contains only the difference between semilocal and exact short-range exchange-energy densities.^18^ Yet the static correlation we want to simulate is clearly a long-range effect. Therefore, our recent scRSLHs add another term with long-range contributions, governed by a switching function *f*_FR_(**r**) that is between 0 and 1 and otherwise closely analogous to *q*_AC_(**r**). The currently best-performing scRSLHs^18,19^ contain an additional correction term in their LMF for delocalization errors in “abnormal open-shell regions” (inspired by aspects of the PSTS LH^35^). During the remainder of this article, we will not pursue RSLHs or scRSLHs, as our uses of machine-learning LMFs so far have focused on LHs and scLHs, as well as on local double hybrids.

We note in passing that the human-designed *q*_AC_(**r**) factors involve so far either a ratio of exact and semilocal exchange-energy densities or a comparison of the normalizations of the exact and a semilocal model exchange hole. In both cases, we find that in certain extreme one-orbital cases, these constructions can erroneously suggest strong correlations due to artificial self-interaction errors in the semilocal energy density or hole function. An example is the electron affinity of the hydrogen atom computed via Koopmans’ theorem from the highest occupied molecular orbital (HOMO) energy of the hydride anion,^18^ but we also see it from LMF shapes for LH25nP (see below) for molecules like HCl or HS.^64^

## 3 Neural-Network LMF, and the LH24n-B95 and LH24n Functionals

Our first use of a neural network (NN) to construct the LMF in an LH^62^ started from the formulation of the t-LMF-based LH20t functional.^67^ The “n-LMF” was trained as a shallow multilayer perceptron (MLP) with only two hidden layers of 64 neurons each (Figure 2). In compact form, an *L*-layer MLP can be written as

3 $$g^{n}\left(r\right)=\phi_{\mathrm{out}}\left(W_{L+1}\phi_{L}\left(...\phi_{1}\left(W_{1}x\left(r\right)+b_{1}\right)...\right)+b_{L+1}\right)$$

where **W**_*i*_ and **b**_*i*_ denote trainable weight matrices and bias vectors for layer *i*, respectively, and each ϕ_*i*_ is a nonlinear activation function. The final activation ϕ_out_ restricts the output to the target interval (e.g., [0,1] or [-1,1]).

**Figure 2:**
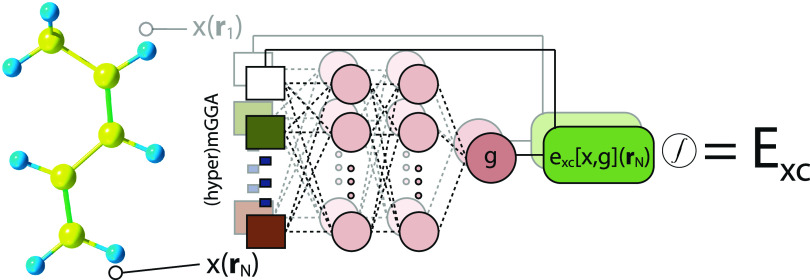
Schematic illustration of machine-learning the LMF in an LH. At each grid point *r*_N_, density-derived (hyper)­meta-GGA ingredients are processed by the same neural network (sharing weights and biases across all points), which outputs the LMF *g*(**r**) that governs the position-dependent EXX admixture. The resulting exchange–correlation energy density *e*_XC_[*x*,*g*]­(**r**_N_) is integrated over the molecular grid to yield the total exchange–correlation energy *E*_XC_.

We settled on seven semilocal input features (**x**(**r**)) for the NN, i.e., the two spin components of the electron density ρ­(**r**), three components of the squared norm of the spin-density gradient, as well as two spin components of local kinetic energy τ­(**r**). While we also tested the EXX energy density as input feature, in the context of that initial work, it did not notably improve performance. As LMFs containing the EXX energy densities complicate somewhat linear-response TDDFT calculations, we preferred the semilocal LMF form for those first attempts. The n-LMF was trained on a relatively small set of BH76 reaction barriers^69,70^ and W4–17 ^71^ atomization energies, with equal weights. That is, essentially no information on static correlation was used, and the n-LMF also remained positive everywhere.

The DC energy contribution of LH20t was based on the B95c meta-GGA correlation functional,^72^ and we initially kept this during the NN optimization of the LMF. This led to the LH24n-B95 functional.^62^ Subsequently, we fixed the n-LMF and replaced B95c by a more flexible B97c-type power-series expansion.^73^ The linear expansion parameters of B97c were optimized for the large GMTKN55 test suite^74^ in conjunction with DFT-D4 dispersion corrections.^14,75,76^ The n-LMF was kept unchanged in that process, but a linear scaling factor was applied to the n-LMF and included in the linear optimization step. This led to the LH24n functional.^62^

The excellent performance of both n-LMF-based LHs for the large GMTKN55 test suite of main-group thermochemistry, kinetics, and noncovalent interactions (NCIs) can be inferred from Table 1. The n-LMF in LH24n-B95-D4 clearly improves performance over LH20t-D4, while the replacement of the original B95c correlation by a more flexibly trained B97c DC contribution improved matters further for LH24n-D4, in particular, regarding NCIs. In fact, at the time LH24n-D4 produced the lowest self-consistent WTMAD-2 value of a rung-4 functional (meanwhile outperformed by our ωLH25tdE-D4 scRSLH^19^ and the LH25nP-D4 scLH^64^ described further below).

**Table 1:** GMTKN55 WTMAD-2 Values for the Usual Subcategories and the Overall Test Suite Given in kcal/mol with Selected Top-Performing Rung-4 Functionals and Skala

method	basic and small	iso and large	barriers	intermol. NCIs	intramol. NCIs	GMTKN55
LH20t-D4	3.11	6.13	4.42	4.94	5.27	4.55
LH24n-B95-D4	2.33	5.33	3.04	3.60	4.02	3.49
LH24n-D4	2.37	4.44	2.86	3.41	3.02	3.10
ωLH25tdE-D4	2.11	2.96	2.49	2.61	3.34	2.64
LH25nP-D4	2.09	2.93	2.44	2.27	2.94	2.47
DM21	1.99	4.64	3.63	6.56	4.16	3.97
ωB97M-V	2.73	4.79	3.40	2.90	4.53	3.53
Skala	2.47	4.22	4.87	2.54	6.37	3.83

Strikingly, in contrast to LH20t where use of a CF is crucial to avoid unphysical Pauli repulsion, in particular in the context of NCIs,^67^ with the n-LMF, all this is achieved without a CF!^62^ While NCIs were not part of the training of the LMF, the final n-LMF must nevertheless have learned a form that helps suppress artifacts from the gauge ambiguity. Gratifyingly, an LMF is a real-space function that can be analyzed in detail for different molecules, for example, in graphical form along the bond axis in a diatomic molecule. An example is shown in Figure 3 for the Ne_2_ system at an interatomic distance of 5.0 bohr. While the t-LMF, which is also provided in Figure 3, has very low values around the bond-critical point (BCP), where it goes exactly to zero by definition, the n-LMF exhibits a flat, broad maximum with a relatively large EXX admixture in that same area. We also include in the plot a so-called x-LMF that was recently designed specifically to suppress gauge artifacts in the LH24x functional.^42^ It is based on the same type of ratio between semilocal and exact exchange-energy densities that enters the *q*_AC_(**r**)-factor of scLHs. Its rationale is to have full EXX admixture in spatial regions where the difference between semilocal and exact exchange-energy density in the middle term of eq 1, i.e., the integrand of the NDC term, takes on unphysical positive values. Notably, the x-LMF goes sharply to 1 at the BCP, i.e., higher but over a narrower area compared to the n-LMF. Both types of LMFs achieve the desired suppression of many of the unwanted gauge artifacts in an LH without the presence of a CF in the functional,^42,62^ and our current research takes these findings into account, e.g., to construct rationally semilocal LMFs with the same properties. We take this as a clear advantage of the described limited use of machine learning for just the LMF of an LH. It definitely provides us with new insights for rational constructions as well, while using data-driven approaches to bridge a knowledge gap in physical constraints for the LMF. Note, however, that due to their lack of sc-corrections, LH24n-B95 and LH24n remain firmly within the usual zero-sum game. We will come back to this (see also Figure 4).

**Figure 3:**
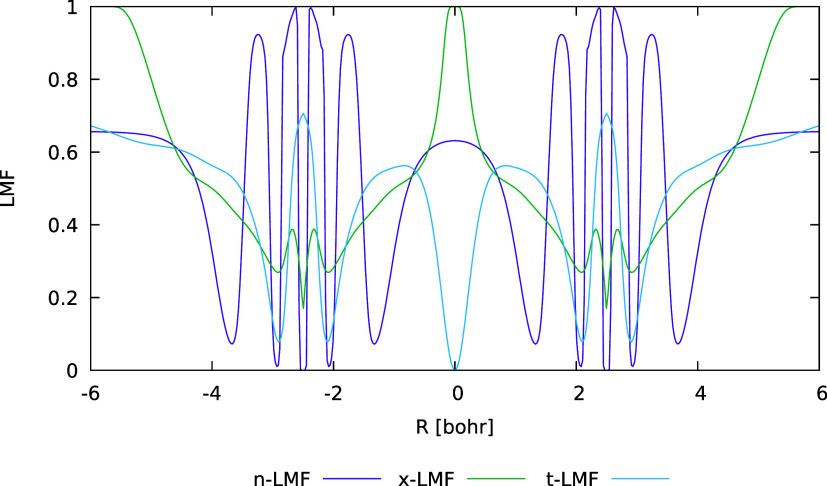
Comparison of LMFs for Ne_2_ at an internuclear distance of 5.0 b.

**Figure 4:**
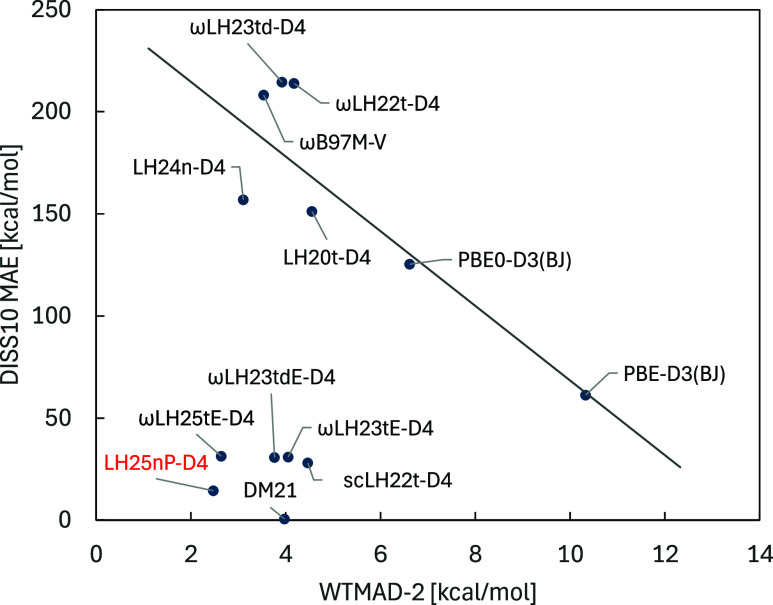
Comparison of plots for different functionals (with dispersion terms included) of the mean-absolute errors for the DISS10 set of spin-restricted covalent-bond dissociation of diatomic main-group dimers in kilocalories per mole as a measure of static-correlation errors against the WTMAD-2 values in kcal/mol of the GMTKN55 suite as a measure of performance for main-group systems that mostly exhibit only weak correlations.

## 4 Using a Neural-Network LMF to Construct the First Local Double Hybrid Functionals

The developments described so far pertain to rung-4 on the famous ladder hierarchy of DFAs,^43^ the “hyper-GGA” rung. On the highest rung-5 we find computationally more demanding functionals with wave function-type correlation contributions that include information from the virtual orbitals. The most prominent subclass on rung-5 are so-called double hybrid (DHs) functionals.^77^ These combine a hybrid functional on the exchange side with a combination of semilocal DFT and fully nonlocal wave function-like contributions on the correlation side. Most often the nonlocal virtual-orbital-dependent contribution in DHs is obtained by second-order perturbation theory applied to the KS orbitals along the lines of Görling–Levy PT2,^78^ the analogue of MP2 in wave function theory. In some cases, spin-component-scaled (SCS) variants are employed.^79^ In others, third-order perturbation has been explored,^80^ or range separation has been applied to the exchange and/or correlation parts.^81^ Several important rung-5 functionals make use of the random-phase approximation within the adiabatic connection fluctuation–dissipation framework and related approaches.^39^

What DHs usually have in common is that their exchange part consists of a global mixing of semilocal and exact exchange, i.e., a GH or possibly an RSH. We asked ourselves if the idea of a position-dependent EXX admixture of an LH, governed by an LMF, can be successfully transferred to a DH, leading to a local double hybrid (LDH).^63^ From the start, we expected that the additional gain in accuracy by position-dependent EXX admixture might be more limited than for an LH, due to the experience that optimized global EXX admixtures of DHs tend to be rather high, often in the 50–75% range. As we wanted to use SCS-MP2-style correlation, we expected the upper part of this range to be relevant. Our starting point was the ωLH22t RSLH functional,^33^ which is based on a t-LMF and features 100% long-range exact exchange. When trying to optimize an LDH based on such a t-LMF, the PT2 part tended to vanish, consistent with the fact that ωLH22t does already perform rather well for typical optimization training test sets. We then proceeded to add a global EXX admixture, i.e., we used an LMF of the form $g\left(r\right)=c_{g}+b\frac{\tau_{W}}{\tau }$ . During optimization, in this case, the spatial dependence of the LMF tended to be optimized out, and we ended with a regular range-separated DH (RSDH), which we named ωDH25. Augmented by D4 correction terms, it provides a GMTKN55 WTMAD-2 value of 2.13 kcal/mol, which appears to be the lowest for a DH used in “gDH” mode,^82^ i.e., when employing the orbitals optimized for the same functional minus the PT2 term to compute all contributions.

In view of these difficulties in optimizing a range-separated LDH with human-designed LMFs, we proceeded to train an n-LMF in post-SCF calculations (based on ωDH25 orbitals) in the presence of PT2 contributions.^63^ That is, the n-LMF was trained on W4–17 atomization energies and BH76 barrier heights, while the other linear parameters of the functional were subsequently optimized for the GMTKN55 test suite. WTMAD-2 values down to 1.88 kcal/mol were obtained in this preliminary post-SCF scheme. This is an improvement of about 0.3 kcal/mol over the corresponding DH with constant EXX admixture, and it is very much at the lower end of values for xDHs in the literature (xDH refers to a flexible protocol where it is allowed to use orbitals from a rather different functional in a post-SCF treatment^83,84^ ), where values down to the 1.72 kcal/mol for the recent DH24 functional^85^ have been achieved. The obtained LMFs were well-structured and consistent with large EXX admixtures in most but not all of space.^63^ The results confirm our assumption that the position-dependent EXX admixture may provide less gain at the DH level than for a regular LH or RSLH. However, we view these results as an important and successful intermediate step in the construction of doubly local double hybrids, where position-dependent admixtures of energy densities are used not only in the exchange part but also for correlation.

## 5 Escaping the Zero-Sum Game by Optimizing a Neural-Network LMF in the Presence of a Strong-Correlation Factor: The LH25nP Functional

For the first LHs with an n-LMF, LH24n-B95 and LH24n,^62^ no attempts were made to incorporate strong-correlation aspects, neither in the training data nor in the LMF setup. We know from human-designed scLHs that the sc-factor reduces locally EXX admixture, in extreme cases of stretched bonds even to the extent that overall locally negative EXX admixtures are observed (see above and Figure 1).^6,22–24^ So far, attempts to train an n-LMF directly to incorporate this behavior by adding strong-correlation test data to the training have not been sufficiently successful. For the moment, we have therefore opted for an intermediate, composite approach, where we took a human-designed, preoptimized, relatively flexible Padé-form *q*_AC_(**r**) function from the scLH23t-mBR-P functional^24^ and trained an n-LMF in its presence. To be able to correct possible inaccuracies of the predetermined *q*_AC_(**r**) in dealing with strong correlations, we (a) trained the n-LMF on a weighted average of W4–17, BH76, diet-GMTKN55 ^86^ data, as well as fractional-spin-error test sets, and (b) we allowed the n-LMF itself to also become negative locally. In view of the larger training data compared to LH24n, the neural network was chosen to be somewhat larger, with three hidden layers of 128 neurons each. Given the use of diet-GMTKN55 data in training, fixed D4 dispersion corrections taken from LH24n-D4 were included during n-LMF training. After training, the linear parameters of both the B97c correlation and the D4 dispersion contributions were reoptimized. The LH25nP scLH^64^ based on this “nP-LMF” uses no CF. We note in passing that in LH25nP the semilocal correlation functional is not multiplied by *q*_AC_(**r**), in contrast to earlier scLHs and scRSLHs with heuristic LMFs. This is the theoretically expected form as the DC contributions should not require enhancement in strong-correlation regions. Numerically, however, it turned out to be advantageous to do it with earlier functionals. Possibly the t-LMFs were insufficiently flexible to account for enough static correlation even after adiabatically connecting the middle term by *q*_AC_(**r**), and one could then benefit from some error compensation between the DC and NDC terms.

As shown in Table 1, LH25nP-D4 achieves a GMTKN55 WTMAD-2 value of 2.47 kcal/mol, the currently clearly lowest value of any rung-4 functional. Given the inclusion of diet-GMTKN55 data in the training procedure, the possibility of overtraining was evaluated by looking at the data obtained after essentially subtracting the training data from those of GMTKN55. Comparison with less parametrized human-designed functionals like ωLH25tdE-D4 suggests only minimal overtraining of the nP-LMF, on the order of ca. 0.15 kcal/mol for GMTKN55. LH25nP-D4 also provides the so far lowest deviations of a rung-4 functional for the W4–11RE set of about 11 000 reaction energies obtained from the W4–11 atomization-energy set.

At the same time, LH25nP gives the lowest errors of our scLHs and scRSLHs for the DISS10 set of spin-restricted covalent-bond dissociation asymptotes, lower than the KP16/B13 functional, comparable to B13 ^64^ (see Section 2 and Figure 1 on how these improvements are achieved by the sc-corrections to the LMF). Only the deep-neural network DM21 functional, which has been explicitly trained on such data, exhibits an even lower value. All of these functionals still have modest unphysical maxima at an intermediate distance in such curves (Figure 1). Also in that respect, LH25nP performs similarly to B13 (and to DM21). Figure 4 plots DISS10 MAEs against GMTKN55 WTMAD-2 values for a number of functionals. Such plots reveal the zero-sum behavior of functionals,^6^ as DISS10 represents static-correlation errors while GMTKN55 represents to a large part weakly correlated main-group energetics. The lower left corner of the plot clusters those functionals that escape to the largest extent the zero-sum game. In this particular representation, the excellent performance of LH25nP-D4 is apparent, as is the best performance of DM21 for the DISS10 set. All functionals in that left bottom corner represent scLHs and scRSLHs. Like some of the best scRSLHs, LH25nP furthermore corrects the unphysical spin-symmetry breaking of certain open-shell transition-metal complexes like MnO_3_ observed with “uncorrected” global, range-separated, local or local range-separated hybrid functionals.^64^

Transferability to organometallic transition-metal reaction energies for LH25nP-D4 is still inferior to LHs or RSLHs based on, e.g., t-LMFs, and 3d transition-metal–ligand bond lengths are also not particularly accurate. This points to the need to widen the training database for such neural-network functionals to include transition-metal systems. On the other hand, LH25nP so far has not been found to exhibit any difficulties regarding SCF convergence, including in transition-metal complexes, in contrast to DM21.^57^

One may ask about the additional computational requirements in practical applications of using a neural-network LMF. We find that upon efficient implementation, the overhead of the n- or nP-LMF tends to be small. Table 2 compares the CPU-time of LMF computation and of one SCF cycle for LH25nP with LH20t as an example for an LH with heuristic LMF and with PBE0 as an example for a GH for a medium-size molecule, acene 10. Even in the initial implementation entered into release 8.0 of Turbomole, n-LMF computation amounts only to about 4% of an SCF cycle. We have meanwhile improved the efficiency of the n-LMF computation so that it amounts only to 0.3% of an SCF cycle in our most recent implementation. The seminumerical computation of the EXX integrals for the LHs with this moderately large basis set is still a bit slower than the analytical calculation used for PBE0. This is known to invert for larger basis sets, due to the lower scaling of the seminumerical integration with basis set and system size. It is clear in any case that computational times are increased very little by employing current neural-network LMFs.

**Table 2:** CPU-Time Comparison in Seconds of LMF Generation and of One SCF cycle for Acene-10 in *D*_2*h*_ Symmetry Using def2-TZVP Basis Sets (54 Atoms and 1174 Basis Functions) for LH25nP with LH20t and PBE0 (the Latter with Analytical Integration of EXX)^a^

functional	LMF computation	SCF cycle	% for LMF
LH25nP	16.6	418.9	4
LH25nP (new)	1.1	406.2	0.3
LH20t	0.0	418.7	0.0
PBE0 (analytical)		360.5	

a Computed on an Intel­(R) Xeon­(R) Silver 4114 CPU@2.20 GHz. Gridsize 3 was used.

## 6 Summary and Outlook

We have discussed an approach to the construction and optimization of exchange-correlation functionals in KS-DFT that uses machine learning in a deliberately limited and transparent way by targeting only the so-called local-mixing function (LMF) in local hybrid and local double hybrid functionals. That is, only the LMF *g*(**r**), i.e., the position dependence of admixture of the exact-exchange (EXX) energy density is trained as a comparably small neural network (n-LMF), within a well-defined structure of the overall functional, where other parameters are obtained in more traditional ways.

The advantage of this approach compared to recent deep-neural network functionals lies in the explainability of the resulting functionals. The LMF *g*(**r**) is a real-space quantity that can be readily visualized for different molecules, providing insight into different aspects. These can then also be used in human-design approaches to the LMF. For example, the first LHs with n-LMFs, LH24n-B95 and LH24n, did already achieve broad accuracy for main-group energetics. And they did this without the need of a calibration function that has previously been assumed to typically be required to mitigate the gauge ambiguity of exchange-energy densities. Graphical comparisons of n-LMFs with human-designed LMFs revealed that the behavior of the LMF near bond-critical points (BCPs) for noncovalent interactions is crucial in this context: while the widely used t-LMF and several other LMFs evaluated in previous work go to zero at the BCP by definition and exhibit very small values around it, the n-LMF and a recent human-designed x-LMF show large values in those regions, in somewhat different ways. We learn from these analyses that (a) the BCP region is the source of the most undesirable gauge artifacts like positive nondynamic correlation energy densities for t-LMFs, and these effects are suppressed by having large EXX admixture in these regions, and (b) relatively large EXX admixtures around the BCP also in covalent bonds are found not at odds with good performance for atomization and reaction energies.

Use of an n-LMF has also, for the first time, allowed the construction of local double hybrid functionals on rung-5, again without the need for calibration functions. Local double hybrids show less improvement over constant EXX admixtures than local hybrids on rung-4. But the obtained n-LMFs are physically intuitive, and the approach may well be extended even to doubly local double hybrid functionals, which are currently being developed in our lab.

We have also discussed the first LH with an n-LMF, LH25nP, that provides the desired escape from the zero-sum game between delocalization and static-correlation errors. This has been achieved by training the n-LMF in the presence of a flexible human-designed strong-correlation factor *q*_AC_(**r**). The latter allows the LMF to become locally negative where appropriate, i.e., when strong correlations are detected in coordinate space. LH25nP-D4 provides the so far best rung-4 performance for main-group energetics (GMTKN55 and W4–11RE) while allowing the proper spin-restricted dissociation of covalent bonds and reducing fractional-spin errors as well as unphysical spin contamination in certain open-shell transition-metal complexes. Again, LMF visualization allows the performance to be understood, while the n-LMF clearly provides improvements in several aspects, including the suppression of gauge artifacts without calibration function.

Important limitations remain and motivate further work. Training so far has emphasized only main-group systems, leading to uneven transfer to transition-metal energetics, spin-state gaps, and metal–ligand bond lengths. Widening the training domain to curated transition-metal and biradical benchmarks is, therefore, a priority. Training of n-LMFs has also not yet addressed the core and asymptotic regions in molecules. These are difficult to optimize in post-SCF training approaches. However, we know from previous work that, e.g., core LMFs are relatively independent of the valence part. This provides promising routes toward combined human-designed/machine-learned LMFs. Further, more technical improvements may involve grid-point training to address quadrature sensitivities and enhance the performance. In general, both integrated observables and local fields may play roles in training. Extensions to range-separated local hybrids are underway and promise further improved accuracy, including the correct asymptotic potential and excellent quasi-particle energies found for purely human-designed functionals like ωLH25tdE.

The approach reported here is positioned between recent black-box deep-neural network functionals like DM21 and Skala and purely human-designed constructions. The focus on the LMF, where the lack of exact constraints has been the most severe obstacle to further improvement, promises and has already shown significant improvements while retaining the explainability of our previous human-designed functionals. Given the proven ability of functionals based on the EXX energy density to escape the zero-sum game, various areas for the further development of broadly applicable exchange-correlation functionals are apparent along these lines, both on rung-4 and on rung-5.
